# Examining the interaction of sex and APOE 4 status in predicting hallucinations relative to delusions in AD

**DOI:** 10.1002/alz70857_099379

**Published:** 2025-12-24

**Authors:** Gayathiri Rajkumar, Marc A Khoury, Mila Valcic, Neda Rashidi‐Ranjbar, Nathan W. Churchill, Luis R Fornazzari, Vincenzo De Luca, Simon J. Graham, David G. Munoz, Tom A. Schweizer, Corinne E. Fischer

**Affiliations:** ^1^ Keenan Research Centre for Biomedical Science, Li Ka Shing Knowledge Institute, St. Michaels Hospital, Toronto, ON, Canada; ^2^ Keenan Research Centre for Biomedical Science, Li Ka Shing Knowledge Institute, St. Michael's Hospital, Toronto, ON, Canada; ^3^ University of Toronto, Toronto, ON, Canada; ^4^ Faculty of Medicine, Department of Neurology, University of Toronto, Toronto, ON, Canada; ^5^ Division of Geriatric Psychiatry, Centre for Addiction & Mental Health, Toronto, ON M5T 1R8, Canada, Toronto, ON, Canada; ^6^ Hurvitz Brain Sciences Program, Sunnybrook Research Institute, Toronto, ON, Canada; ^7^ University of Toronto, Torono, ON, Canada; ^8^ Temerty Faculty of Medicine, Department of Laboratory Medicine and Pathobiology, University of Toronto, Toronto, ON, Canada; ^9^ Division of Pathology, St. Michael's Hospital, Toronto, ON, Canada; ^10^ Temerty Faculty of Medicine, Division of Neurosurgery, University of Toronto, Toronto, ON, Canada; ^11^ Temerty Faculty of Medicine, Department of Psychiatry, University of Toronto, Toronto, ON, Canada

## Abstract

**Background:**

The APOE4 allele is a significant genetic risk factor for Alzheimer's disease (AD). Prior work from our group suggests that among neuropathologically verified AD patients, homozygous APOE4 status increases psychosis risk in females compared to heterozygotes, non‐carriers, and males but only in the presence of Lewy Body (LB) pathology. This study examines the effects of APOE4 carrier status (homozygous, heterozygous, or non‐carrier) and sex on the visual hallucinations relative to auditory hallucinations and delusions in AD. We hypothesize that female‐specific findings will be greater in visual hallucinations compared to auditory hallucinations and delusions given they are a proxy of LB pathology.

**Method:**

Patient data was extracted from the NACC database. The sample was limited to patients with an etiologic diagnosis of AD. Psychosis was defined based on Neuropsychiatric Inventory subscores for delusions and hallucinations. We analyzed data separately for female APOE4 homozygotes, heterozygotes and non‐carriers as well as male APOE4 homozygotes, heterozygotes and non‐carriers using a binary logistic regression. Covariates, including age, education, Montreal Cognitive Assessment (MoCA) scores, and Mini‐Mental State Examination (MMSE) scores, were controlled for in all analyses.

**Result:**

When pooling males and females together, APOE4 homozygotes had a significantly greater likelihood of auditory hallucinations compared to heterozygotes and delusions compared to non‐carriers (*p* < .05). When comparing female APOE4 homozygotes relative to female heterozygotes and non‐carriers, homozygous individuals displayed a greater likelihood of hallucinations compared to non‐carriers (*p* < 0.05). Among males there were significant differences in hallucinations overall, visual and auditory among homozygotes relative to heterozygotes and no differences with non‐carriers (*p* < .05).

**Conclusion:**

Findings suggest that APOE4 homozygosity increases psychosis risk among males relative to heterozygotes and females relative to non‐carriers, with effects being limited to hallucinations. Pooled analyses in both sexes showed effects of APOE4 mainly in delusions and auditory hallucinations. This data suggests the sex effects of APOE4 may be stronger for hallucinations relative to delusions though further studies in larger samples are needed to corroborate this finding. It should also be noted that these analyses did not adjust for LB pathology.

